# Prevalence and local factors associated with radiographic bone loss in primary dentition: A retrospective study

**DOI:** 10.1002/jper.70075

**Published:** 2026-02-26

**Authors:** Murilo C. Paraluppi, Camila S. Stolf, Bianca Mendes, Marcio Z. Casati, Karina G. Silvério, Enilson A. Sallum, Renato C. V. Casarin, Mabelle F. Monteiro

**Affiliations:** ^1^ Periodontics Division Department of Prosthodontics and Periodontics Piracicaba Dental School University of Campinas Piracicaba São Paulo Brazil

**Keywords:** adolescent, alveolar bone loss, child, periodontitis, primary teeth

## Abstract

**Background:**

This study aimed to evaluate the radiographic bone level in primary molars in a Brazilian population, define the prevalence of bone loss, and identify the local factors associated with early bone alterations.

**Methods:**

Radiographs of children who attended Piracicaba Dental School (Brazil) between 2014 and 2020 were evaluated. Two calibrated examiners measured the distance between the cementoenamel junction and the bone crest (CEJ‐BC) in bitewing radiographs, and CEJ‐BC > 2 mm represented bone loss. Local factors in the measured sites were screened. Multiple linear and logistic regressions were used in the analysis.

**Results:**

828 bitewing radiographs from 527 patients (8.27 ± 1.71 years) were evaluated. The mean CEJ‐BC distance was 1.12 ± 0.40 mm (0.0–3.4 mm). 85 sites were positive for bone loss, with a prevalence of 2.62% at site and 13.28% (70 individuals) at patient levels. Male patients, increasing age, maxilla, first primary molars, and local factors were related to a higher CEJ‐BC. Pulpectomies, fillings, and adjacent tooth eruption were positively associated with bone loss, and those statistically significant local factors were present in 42.4% of the sites with bone loss.

**Conclusion:**

The prevalence of radiographic bone alterations in primary molars was 13.28%. The CEJ‐BC distance and bone loss were positively associated with local factors (58.8% of the cases); however, 41.2% of the sites positive for bone loss did not present the local factors evaluated in this study.

**Clinical relevance:**

1. **Scientific rationale for this study**: Bone loss can be present in children and adolescents, and radiographic evaluation could help to elucidate its prevalence and the factors associated with its occurrence.
2. **Principal findings**: A prevalence of 13.28% bone loss was found at the patient level in primary molars in a Brazilian population, and local factors were positively associated with bone alterations. Despite some cases diagnosed with bone loss being related to local factors, 41.2% had no association with the local risk factors evaluated in the study.
3. **Practical implications**: The findings regarding bone loss prevalence in primary dentition highlight the importance of careful radiographic examination in children and adolescents as an initial tool to diagnose periodontitis at early stages and to prevent severe damage.

**Plain language summary:**

This study analyzed x‐rays of Brazilian children to assess bone loss in primary molars. It aimed to determine how common bone loss is and to identify local factors linked to the associated bone loss. Researchers reviewed 828 x‐rays from 527 children (average age 8.27 years) who visited Piracicaba Dental School between 2014 and 2020. Bone loss was identified when the distance between the cementoenamel junction and bone crest (CEJ‐BC) was greater than 2 mm. The results showed an average CEJ‐BC distance of 1.12 mm, with 85 areas showing bone loss. The bone loss prevalence was 2.62% per site and 13.28% per patient. Factors linked to higher bone loss included being male, older age, having teeth in the maxilla, first primary molars, and specific local factors. Procedures such as pulpectomies, fillings, and nearby tooth eruptions were associated with bone loss; however, 41.2% of cases identified with bone loss did not present the local factors evaluated in the study. The study concluded that 13.28% of children showed bone changes in their primary molars, with bone loss linked to local factors and other conditions.

## 1 INTRODUCTION

Factors related to genetics, individual inflammatory and microbiological profiles, and even environmental factors influence the occurrence of periodontitis, making it difficult to establish a precise chronology of events involved in its onset. In some cases, advanced periodontal damage and rapid progression rates can be found in young patients, including children and adolescents, leading to early tooth loss when untreated.^1^ Considering the difficulty in predicting which individuals will develop the disease in the future, the most accessible way to avoid major periodontal losses would be to focus on disease prevention and early diagnosis when damage to the periodontium is still limited and more easily treated. Therefore, obtaining epidemiological data on bone loss in children and its possible risk factors is fundamental but yet unexplored.

Many studies have focused on identifying clinical and molecular factors associated with the early development and rapid progression rate of the disease. Factors such as altered microbiota, inflammatory response pattern, family correlations, and individual susceptibility^2^, ^3^, ^4^, ^5^, ^6^, ^7^ have already been described as being capable of influencing the disease occurrence. Nevertheless, no diagnostic tool or marker can predict bone loss, periodontitis activity, and susceptible sites and individuals. Thus, clinical and radiographic evaluation is still essential to diagnose the disease precociously and to identify susceptible individuals.

Given the few studies on children, the prevalence of clinical attachment loss in young people is not well defined.^7^, ^8^ The fact that the disease can be present in primary and mixed dentition, in which physiological changes occur quickly and dynamically, adds complexity to diagnosing periodontitis.^9^ The chronic nature of tissue destruction during periodontitis also requires a period of occurrence to be identified in clinical and radiographic examinations. Besides, the diagnosis of periodontitis is hampered by the lack of routine periodontal examination in children focusing on identifying clinical attachment loss, including periodontal probing and looking for initial bone loss in interproximal radiographs.

A recent study evaluating a radiographic database of children and adolescents between 1 and 15 years of age (mean age 6.46 ± 2.38) observed a prevalence of radiographic bone resorption of 3.52% among the evaluated children, but 81% of the sites were significantly associated with local factors, indicating that only 19% of these cases would be related to periodontitis.^10^ However, this study did not perform any multivariate analysis to identify the relationship between each variable and the bone alterations. In addition, it was carried out in a Caucasian population in which the prevalence of periodontitis is lower than in populations from the African and American continents.^8^, ^11^ According to those reviews conducted in children and the young population,^8^, ^11^ the prevalence of aggressive periodontitis was reported as 0.1%–0.5% in Europe, 1%–5% in Africa, and 0.3%–3.7% in Brazil. Thus, assessing the prevalence of radiographic bone loss in children in countries where a higher prevalence of disease is observed is essential. This approach would favor early diagnosis and understanding the aspects related to the onset and susceptibility to the disease.

Thus, this study aimed to evaluate the radiographic bone level in primary dentition in a Brazilian population, define the prevalence of bone loss, and to identify the local factors associated with early bone alterations.

## 2 MATERIALS AND METHODS

This retrospective study was performed at Piracicaba Dental School after approval by the local ethical committee (protocol number 48784521.5.0000.5418) and study registration on the Brazilian Registry of Clinical Trials (ReBEC) (identifier RBR‐53gfpy9). The studied data were collected and analyzed between September 2022 and September 2023.

All radiographs evaluated in this study were taken from the radiographic database of the Piracicaba Dental School's undergraduate clinic. All patients were evaluated anonymously; each participant was identified by a unique numerical code during data acquisition, and the examiners had no access to their identities. All radiographs were obtained using a digital system used by the FOP‐UNICAMP graduation clinic (Apixia PSP Digital Scanner, USA), which guarantees high‐quality images and minimal distortion.

### 2.1 Inclusion criteria

The study included interproximal radiographs of primary molars from patients of both sexes, aged between 3 and 15 years at the time of bank image screening (between 5 and 13 years of age at the time of the included radiograph), who presented radiographs taken between 2014 and 2020. In the case of patients with multiple radiographs, the most recent of each region was included as a measurement reference for the analysis.

### 2.2 Exclusion criteria

Bitewing radiographs excluded from the evaluation: (i) were not angulated perpendicular to the tooth, (ii) did not present good sharpness.

Individual sites were excluded from the analysis when: (i) the cementoenamel junction (CEJ) or the bone crest (BC) were not visible, (ii) overlapping structures that made the evaluation unfeasible were present, (iii) primary molars were exfoliating with extensive root resorption (the root completely reabsorbed or reabsorbed and completely detached from the periodontium).

### 2.3 Radiographic evaluation

Before inclusion in the study, radiographic images were previously screened for minimum quality standards to guarantee that the assessment of bone levels could be performed consistently.

Measurements were performed similarly to those described by Wylleman et al.^10^ Two examiners (MCP and BM) analyzed the radiographs, measuring the distance between the CEJ and the BC using the ImageJ software (version v1.53k). The radiographic film dimension was used to calibrate the measurements in ImageJ before the CEJ‐BC distances were taken. Before the study measurements, each examiner was calibrated to standardize the evaluation criteria. The examiners evaluated 40 radiographs, twice each, to establish agreement of the intra‐examiner and inter‐examiner measurements. The correlation coefficients for the CEJ‐BC distance were 0.797 for inter‐examiner and 0.97 for intra‐examiner, with examiner 1 and 0.94 for examiner 2, respectively.

**FIGURE 1:**
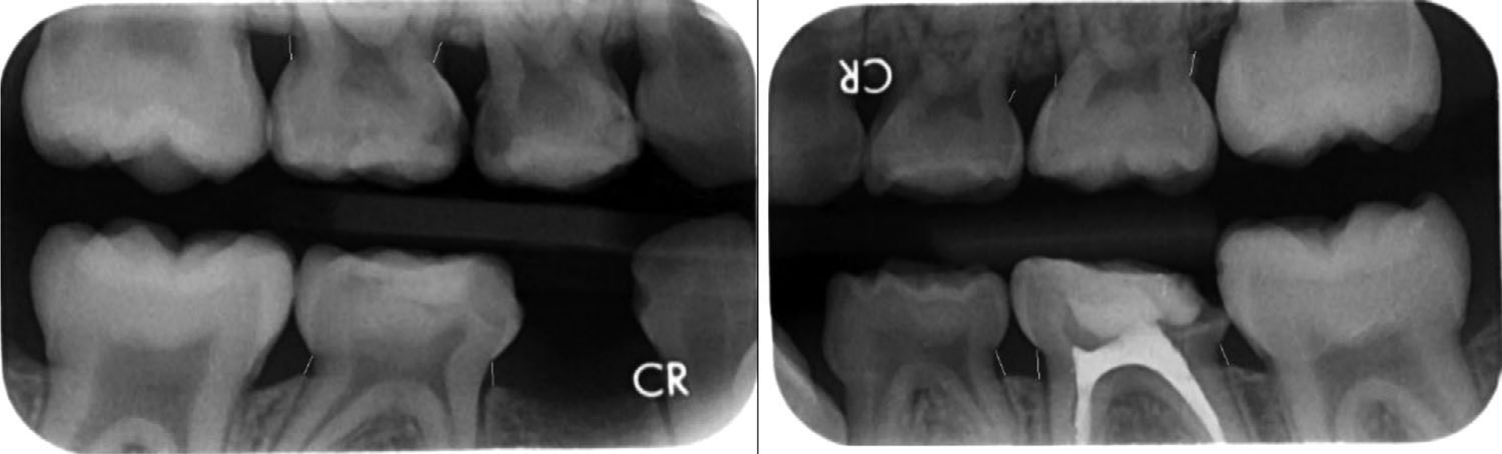
Radiographs of an 8‐year‐old patient describing digital measurements by tracing a cementoenamel junction and bone crest (CEJ‐BC) line using the Image J software.

The measurement was made by drawing a straight line between the CEJ and BC, as described in Figure 1. The evaluation considered a standard of normality of up to 2 mm^9^, ^10^; thus, measurements greater than 2 mm between CEJ‐BC were described as positive for bone loss. To control possible measurement errors, all sites where one examiner measured a distance greater than 1.9 mm and sites with a difference between examiners greater than 0.5 mm were reassessed by both examiners together.

Additionally, to the CEJ‐BC measurement, the radiographs were evaluated regarding the local factors in areas close to the examined sites, such as caries, restorations, stainless steel crowns, teeth in exfoliative processes (primary teeth with more than two‐thirds of root resorption while still preserving some periodontal attachment), teeth in eruption, pulp alterations, use of orthodontic appliances, or other external factors that may influence the physiology of the bone resorption and neoformation process.

The presence of the lamina dura was not evaluated in the study, as it could not be evaluated precisely in most cases, which made the measurement standardization difficult and could result in subjective analysis.

### 2.4 Statistical analysis

Demographic characteristics of the individuals were evaluated using descriptive analysis. The average CEJ‐BC distance of the two examiners at each site was used in the analysis. The differences in the mean CEJ‐BC distance for sites presenting or not the local factor was tested using the Mann–Whitney test. The difference in the percentage of sites presenting bone loss or not for each local and demographic factor was tested using the Chi‐square test. A multiple linear regression model with adjustment for repeated patient measurements was used to identify the contribution of the variables evaluated in the study on the CEJ‐BC distance. Multiple logistic regression, considering the presence or absence of bone loss as the dependent variable and the other study variables as independent was used to identify the factors related to bone loss. All analyses were performed using the software JPM Pro (14.0.0), considering a significance level of 5%.

## 3 RESULTS

**FIGURE 2:**
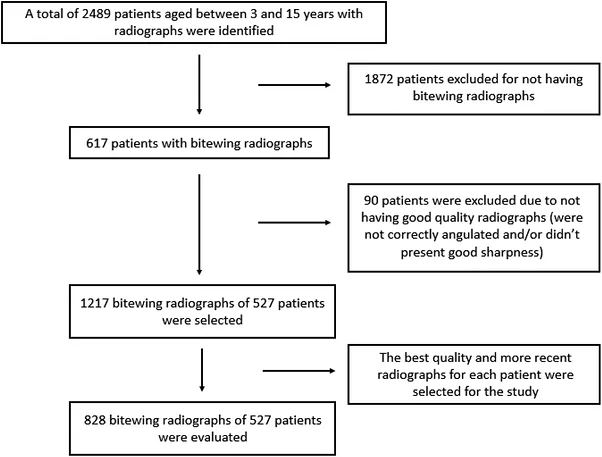
Patients' inclusion‐exclusion flowchart.

The study included 527 patients, 828 bitewing radiographs, and 3245 sites. Figure 2 describes a flowchart of the patients’ enrollment.

**FIGURE 3:**
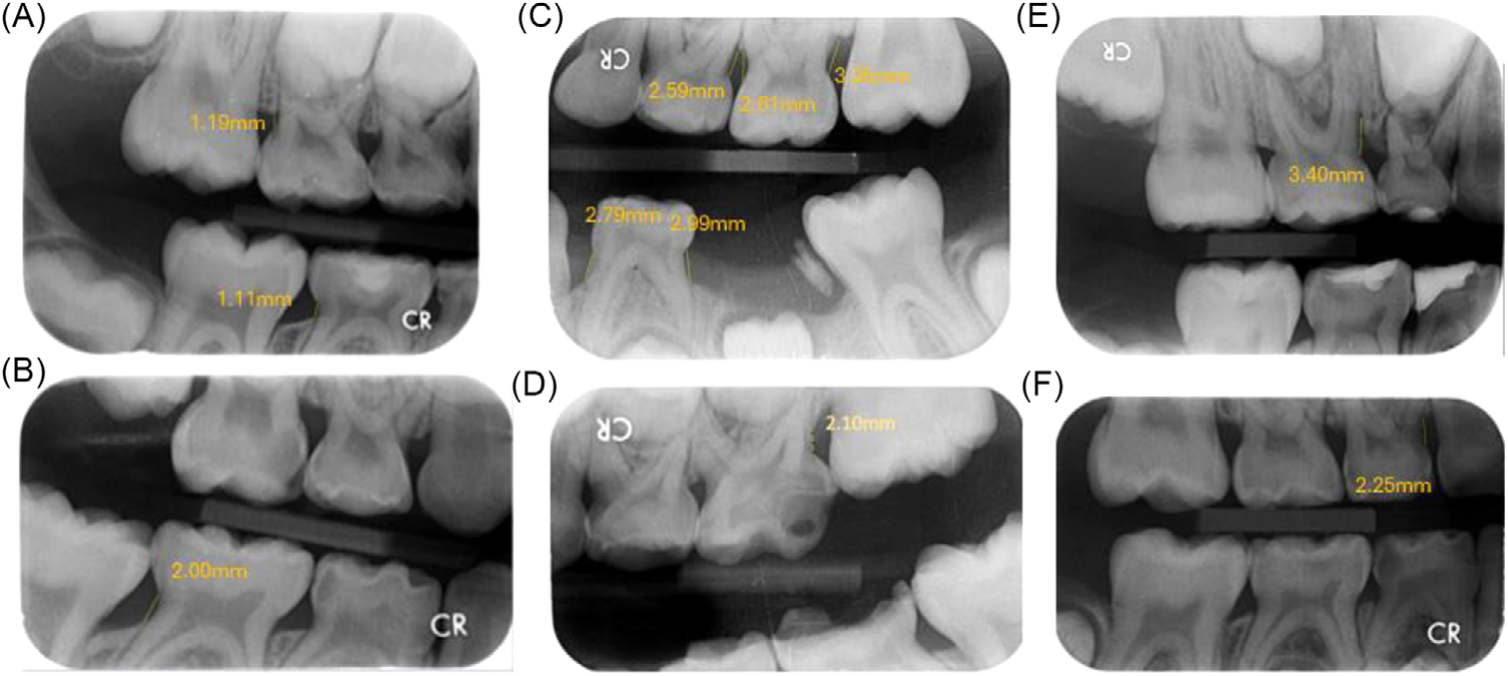
(A) Radiograph of a patient (9 years old) without radiographic bone loss showing measurements around 1.1 mm and 1.2 mm. (B) Example of a borderline radiographic cementoenamel junction and bone crest (CEJ‐BC) distance (2 mm) in a patient with 6 years old. (C) Patient (9 years old) showing radiographic bone loss in multiple sites and presenting measurements between 2.59 mm and 3.26 mm. (D) Patient (10 years old) showing a discrete increase in CEJ‐BC distance (2.10 mm) in a site associated with a carious lesion. (E) Example of a patient (10 years old showing radiographic bone loss on a site without associated local factors (3.40 mm). (F) Patient (10 years old) showing radiographic bone loss without surrounding local factors (2.25 mm).

The included patients' mean age was 8.27 ± 1.71 years (5‐13) and 48.2% were female. Of 527 evaluated patients 70 (13.28%) had at least one positive site for bone loss (i.e., CEJ‐BC distance greater than 2 mm). Of the evaluated sites, 85 (2.62%) were positive for bone loss. The mean CEJ‐BL distance for all patients was 1.12 ± 0.40 mm (0–3.4 mm). A higher CEJ‐BL distance was observed for bone loss sites (2.31 ± 0.30 mm) than for sites without bone loss (1.09 ± 0.35 mm) (*p* < 0.001). The description of the clinical scenarios observed during measurement is represented in Figure 3.

Table 1 describes the influence of clinical and demographic variables on the CEJ‐BC distance. The presence of local factors “pulpectomy”, “caries”, “exfoliating tooth”, and “erupting adjacent tooth”, lower jaw sites, and male patients were associated with higher CEJ‐BC.

**TABLE 1:** Impact of demographic and local factors on the radiographic cementoenamel junction and bone crest (CEJ‐BC) distance.

	CEJ‐BC (mm ± SD)	*p*‐value
**Pulpectomy**		
No (*n* = 3183)	1.12 ± 0.40	0.001
Yes (*n* = 62)	1.29 ± 0.46	
**Caries**		
No (*n* = 2759)	1.11 ± 0.40	<0.0001
Yes (*n* = 486)	1.18 ± 0.40	
**Fillings**		
No (*n* = 2625)	1.11 ± 0.40	0.055
Yes (*n* = 620)	1.16 ± 0.42	
**Tooth exfoliating**		
No (*n* = 3052)	1.11 ± 0.40	<0.0001
Yes (*n* = 193)	1.33 ± 0.42	
**Adjacent tooth exfoliating**		
No (*n* = 3209)	1.12 ± 0.40	0.049
Yes (*n* = 36)	1.23 ± 0.43	
**Adjacent tooth erupting**		
No (*n* = 3193)	1.12 ± 0.40	<0.0001
Yes (*n* = 52)	1.47 ± 0.61	
**Sex**		
Female (*n* = 1542)	1.08 ± 0.39	<0.0001
Male (*n* = 1703)	1.15 ± 0.40	
**Jaw**		
Maxilla (*n* = 1559)	1.19 ± 0.40	<0.0001
Mandible (*n* = 1686)	1.05 ± 0.40	
**Tooth region**		
Mesial (n = 1305)	1.11 ± 0.44	0.200
Distal (*n* = 1940)	1.12 ± 0.37	
**Tooth**		
1^st^ primary molar (*n* = 1165)	1.18 ± 0.37	<0.0001
2^nd^ primary molar (*n* = 2080)	1.09 ± 0.41	

Table 2 represents the adjusted multiple regression model for CEJ‐BC distance. The model was produced with 3245 observations, and the adjusted R^2^ was 0.31. The only variable removed from the model after adjustment was the tooth region; thus, mesial or distal tooth surfaces do not interfere with the bone level. Male patients, the increase in age, maxilla, first molars, and the presence of caries, pulpectomy, tooth exfoliating, adjacent tooth exfoliating, and tooth erupting tend to present a higher CEJ‐BC.

**TABLE 2:** Adjusted multiple regression using the restricted maximum likelihood method evaluating the effect of clinical and demographic factors on the dependent variable cementoenamel junction and bone crest (CEJ‐BC) distance.

	Estimate	95% CI	Std Error	DFDen	t Ratio	Prob > |t|
Intercept	1.49	1.35–1.64	0.07	1443.00	20.18	<0.0001
Sex [Male]	0.03	0.02–0.05	0.01	440.20	3.56	0.0004
Age	0.01	0.00–0.03	0.01	548.00	2.10	0.0363
Jaw [Maxilla]	0.07	0.06–0.08	0.01	3030.00	10.66	<0.0001
Tooth [2^nd^ primary molar]	0.05	0.03–0.06	0.01	2967.00	7.03	<0.0001
Pulpectomy [Yes]	0.07	0.02–0.12	0.02	3234.00	2.92	0.0035
Caries [Yes]	0.04	0.02–0.06	0.01	3233.00	3.94	<0.0001
Fillings [Yes]	0.03	0.02–0.05	0.01	3061.00	3.78	0.0002
Tooth exfoliating [Yes]	0.09	0.06–0.12	0.02	3094.00	5.85	<0.0001
Adjacent tooth exfoliating [Yes]	0.07	0.00–0.13	0.03	3216.00	2.09	0.037
Adjacent tooth erupting [Yes]	0.20	0.15–0.25	0.03	3145.00	7.66	<0.0001

The data were also evaluated according to the presence or absence of bone loss. The BL > 2 mm limit was used to determine the presence of bone loss. The frequency of each clinical and demographic variable was described for sites that did or did not have bone loss (Table 3). Higher bone loss was observed in the maxilla, mesial surfaces, male patients, and in sites with pulpectomies, fillings, and adjacent tooth eruption. From the 85 sites presenting bone loss, 35 (41.2%) did not present the associated local factor evaluated in the study, and 36 (42.4%) presented pulpectomies, fillings, and/or adjacent tooth erupting (the statistically significant local factors based on multiple logistic regression – Table 4). The other 14 sites were associated with local factors without statistical significance for bone loss based on the multiple regression.

**TABLE 3:** Frequency of detection of local factors in sites with and without bone loss.

	Without bone loss (*n* = 3160)	With bone loss (*n* = 85)	*p*‐value
Pulpectomy [*n* (%)]	56 (1.77)	6 (7.06)	0.006
Caries [*n* (%)]	470 (14.87)	16 (18.82)	0.32
Fillings [*n* (%)]	594 (18.80)	26 (30.59)	0.01
Tooth exfoliating [*n* (%)]	184 (5.82)	9 (10.59)	0.09
Adjacent tooth exfoliating [*n* (%)]	34 (1.08)	2 (2.35)	0.33
Adjacent tooth erupting [*n* (%)]	43 (1.36)	8 (9.14)	<0.001
Sex [*n* (%) Male]	1647 (52.12)	56 (65.88)	0.011
Jaw [*n* (%) Maxilla]	1506 (47.66)	53 (62.35)	0.007
Tooth region [*n* (%) Mesial]	1352 (39.62)	53 (62.35)	<0.001
Tooth [*n* (%) 1^st^primary molar]	1140 (36.08)	25 (29.41)	0.19

**TABLE 4:** Multiple logistic regression that considered the presence or absence of radiographic bone loss as a dependent variable.

	Estimate	Std Error	ChiSquare	Prob > ChiSq	Odds Ratio	CI 95%
Intercept	−3.33	0.89	14.13	0.000		
Sex [Male]	0.28	0.12	5.49	0.019	1.74	1.09–2.77
Age	0.24	0.08	9.81	0.002	1.27	1.09–1.47
Jaw [Maxilla]	0.35	0.12	8.49	0.004	2.00	1.25–3.21
Tooth region [Mesial]	0.43	0.12	13.43	0.000	2.38	1.49–3.79
Tooth [1^st^ primary molar]	0.02	0.13	0.01	0.903	1.03	0.62–1.71
Pulpectomy [Yes]	0.65	0.23	7.72	0.006	3.68	1.46–9.23
Caries [Yes]	0.24	0.15	2.62	0.105	1.60	0.90–2.84
Fillings [Yes]	0.35	0.13	7.64	0.006	2.00	1.22–3.27
Tooth exfoliating [Yes]	0.21	0.2	1.16	0.281	1.56	0.70–3.29
Adjacent tooth exfoliating [Yes]	0.38	0.38	1.03	0.311	2.14	0.49–9.44
Adjacent tooth erupting [Yes]	1	0.22	21.42	<0.0001	7.35	3.16–17.14

Multiple logistic regression was used to detect how the variables are jointly related to bone loss (Table 4). Being a male patient, increased age, maxilla, mesial surface, pulpectomy, fillings, and an adjacent tooth erupting were positively associated with bone loss. Sites adjacent to an erupting tooth had a 7.35 times higher chance of presenting bone loss, teeth presenting with pulpectomy had a 3.68 times higher chance of presenting bone loss, and an increase of one year of age represented a 1.27 times higher chance of presenting bone loss.

As the literature has reported differences in bone loss associated with periodontitis in 1^st^ primary molars and 2^nd^ primary molars,^9^, ^12^ an additional analysis considering the frequency of bone loss associated with no local factors in each primary molar was performed. The study identified that 64% of the 1^st^ primary molars with bone loss presented no associated local factor, while 31.67% of the 2^nd^ primary molars with bone loss presented no associated local factor. Thus, 1^st^ primary molars presented a higher frequency of bone loss associated with no local factor than 2^nd^ primary molars (*p* = 0.0006).

## 4 DISCUSSION

Besides being less frequent compared with adults, periodontitis can also be identified in children and adolescents. However, little is known about the prevalence of bone loss in children, the factors that interfere with the beginning of periodontal attachment loss, and its risk assessment. In addition, diagnosis does not usually occur in this age group, due to both the lack of adequate clinical and laboratory methods and the lack of professional focus on identifying bone loss in children. This study used a radiographic database to identify bone level alterations and associated local factors. To the best of our knowledge, this was the first study to assess the prevalence of radiographic bone loss in primary dentition in a population with a high prevalence of periodontitis, such as the Brazilian population presenting a high percentage of African American descendants. The patients, even at low ages (5 to 13 years of age), had a prevalence between 2.62% and 13.28% of radiographical bone loss at the site and patient levels, respectively, and local factors significantly impacted bone level and the presence of bone loss in some cases.

In an article by Wylleman et al.,^10^ using a similar study design and measurement criteria, children were radiographically evaluated in the primary dentition and showed a prevalence of 3.52% of radiographic bone loss at least in one site, with a mean CEJ‐BC of 0.93 ± 0.37 mm (0.07–2.88 mm). This frequency of bone alteration was lower than that identified in the present study, in which the identified prevalence of bone loss at the patient level was 13.28% (mean distance of CEJ‐BC of 1.12 ± 0.40 mm for the all sites, a range of 0.0–3.4 mm, CEJ‐BL for bone loss sites of 2.31 ± 0.30 mm, and 1.09 ± 0.35 mm for sites negative for bone loss). Differences in BL frequency across studies may be related to demographic differences between the two populations.

Wylleman's study^10^ evaluated the Belgian population, which is predominantly Caucasian, with an estimated black population of 3.6%, according to a 2019 estimate from Belgium.^13^ The Brazilian population, on the other hand is marked by high miscegenation and a higher percentage of African descendants, as demonstrated in the last population census done in 2022,^14^ in which 45.5% of the Brazilian population declared themselves as brown and 10.2% as black, while white people represented 43.5% of the population. This difference corroborates the epidemiological data of periodontitis in children and young individuals^8^, ^11^ that describes a lower prevalence of periodontitis in Caucasians compared with the African descendant population (0.1%–0.5% in Europe, 1%–5% in Africa, and 0.3%–2.0% in South America). The authors present studies from Brazilian populations, with prevalence rates ranging from 0.3% to 3.7%; in populations of young adults, the prevalence reached 5.5%. Despite this known difference between populations, neither Wylleman and collaborators^10^ nor the present study evaluated the ethnicity of the included population, and the relationship between bone alterations and ethnicity in both studies remains speculative.

In addition to ethnicity, age can affect this result. The bone level was related to increasing age, and there is a difference between the mean age of the children in the present study, with a mean age of 8.12 ± 1.60 years (between 5 and 13 years), and in Wylleman et al.,^10^ 6.46 ± 2.38 years (between 1 and 15 years). This difference could explain part of the divergence in the mean CEJ‐BL distance, which is slightly higher in the present study. Since periodontitis is a chronic and cumulative disease, older individuals are more likely to have clinical signs of the disease, so this could contribute to a greater prevalence of bone loss. Likewise, Susin & Albandar^15^ evaluated the prevalence of aggressive periodontitis (AgP) in a similar population, and a positive correlation between increasing age and AgP diagnosis was identified, in which subjects from the age group from 25 to 29 years presented 9.9% of AgP diagnosis vs. 2.5% from the 14 to 19 year age group. Even though it is less prevalent at a young age, care policies targeting diagnosis and prevention in young individuals are still necessary to reduce periodontitis damage.

Regarding local factors influencing the periodontium, it was observed that the bone level was positively impacted by tooth eruption and exfoliation. These data corroborate the hypothesis that the dynamics of bone resorption and neoformation associated with the transition from primary to permanent dentition may hinder the diagnosis of pathological periodontal alterations. This association was observed when the evaluated tooth was exfoliating and when the adjacent tooth was erupting. Those conditions are transitory and should be clinically assessed and monitored over time to determine whether they represent a temporary adaptation of the periodontium or pathological bone loss, and thus to enable early diagnosis of bone loss and periodontitis. In this study, all erupting or exfoliating teeth located adjacent to a measurement site were considered a local factor, categorized as “adjacent tooth erupting” or “adjacent tooth exfoliating”, and there was no distinguishment regarding which tooth (primary or permanent) was erupting. Furthermore, an erupting tooth is usually associated with an alteration in the occlusal plane; however, it was not considered an individual factor in the analysis due to the relationship between variables and the importance of a clinical evaluation to directly describe the occlusal condition.

Carious lesions and upper jaw sites were also associated with a higher bone level. The bone quality of the maxilla and mesial and distal furcation in the upper molar can locally influence the bone levels. At the same time, carious lesions can be a local retention of biofilm that stimulates local inflammation and bone resorption. The association between caries and periodontitis in young subjects was also described in a population 13‐29 years old by Costa et al.,^16^ in which untreated caries was grouped with moderate CAL and PPD in the statistical analysis, suggesting that the onset and progression of these two conditions may occur early in life concomitantly.

Despite the importance of local factors and the model's significance, it is interesting to note that the adjusted R^2^ obtained in the linear regression was 0.31, which means that the model explains only 31% of the bone level variability based on the included local and demographic factors. Thus, there is a need for a broader assessment of clinical, microbiological, and immunological evaluations, family history, and genetic factors to fully understand the changes in bone levels in the young population. The impact of family on bone loss and periodontitis, for example has been evaluated in the literature. Tabaa and colleagues^17^ demonstrated that siblings affected by grade C molar–incisor pattern periodontitis exhibit consistent distributions of bone loss and symmetry in periodontitis presentation across the primary and permanent dentitions within families, reinforcing the role of familial susceptibility.^17^ Furthermore, a family history of periodontitis has been shown to affect the periodontal inflammatory response pattern^18^ and early‐life oral microbiological colonization,^4^ thereby influencing susceptibility to periodontitis. Thus, a complex panel should be evaluated to elucidate bone loss at early ages.

Regarding bone loss, local risk factors should be evaluated to understand whether the observed bone loss could be related to periodontitis. Once local factors have been positively associated with bone loss (OR = 7.35 and 3.68 for adjacent tooth erupting and pulpectomy, respectively), these factors may influence the bone level, and the bone alteration could be unrelated to periodontitis. Despite its relevance, this situation did not represent most cases in this study. Only 42.4% (36) of sites presenting bone loss were associated with statistically significant local factors vs. 41.2% (35) with no association with local risk factors evaluated in this study. These findings differ from those of Wylleman et al.,^10^ in which 81.25% of sites with bone loss were positively associated with local risk factors. Therefore, it is more plausible that the bone loss identified in the present study is more often representative of early signs of periodontitis. However, this hypothesis cannot be confirmed without clinical evaluation of the patients, including periodontal probing, clinical attachment level evaluation, and bleeding on probing, which are a fundamental part of periodontal diagnosis.

Another interesting observation from the study is that the 1^st^ primary molars identified with bone loss presented a higher frequency of sites with no associated local factor (64%) than 2^nd^ primary molars (31.67%), and thus those sites could have a higher probability of being related to periodontal disease. This factor aligns with evidence that 1^st^ primary molars are more frequently and more severely affected by periodontitis‐related bone loss than 2^nd^ primary molars,^9^ and this information can drive early clinical diagnosis.

Furthermore, other local factors that were not evaluated in the study due to the absence of clinical evaluation or limitations inherent in a cross‐sectional retrospective evaluation from a bitewing radiographic database (such as open contact, calculus, malocclusion, missing tooth, misalignment, hypereruption, and ankylosis) can also potentially influence bone loss, impact these percentages, and it is a limitation of this study.

Some aspects that could facilitate the differentiation between bone loss related to periodontal disease or to other reasons, such as the presence of an intact cortical bone on the crest and the presence of radiolucency on the furcation were not evaluated in the study due to the difficulty in standardizing those measures on the database images and the influence of radiographic conditions on their identification. However, their evaluation is fundamental in association with clinical examinations when diagnosing early cases of periodontal loss. Thus, future studies focusing on radiographic identification of periodontitis should obtain high‐quality, well‐positioned radiographs to improve the observation of these structures. Furcation radiolucency and bone crest blurring are the most precocious signs of bone loss, and the evaluation of those regions in good‐quality radiographs is a key point of diagnosis of early signs of periodontitis in primary dentition.

Although all the variables mentioned make the early diagnosis of periodontitis somewhat complex, and several studies suggest a low prevalence in children, there is still another little‐explored field in this matter: the search for radiographic alterations in pediatric patients tends to focus mainly on caries diagnosis, justified due to the high prevalence of this disease. However, it is possible that early signs of periodontitis (characterized by incipient interproximal bone loss) would be diagnosed more frequently if these radiographs were carefully evaluated for periodontal changes and monitored. This idea is illustrated by Miller et al.,^9^ who retrospectively evaluated radiographs of adolescents diagnosed with periodontal loss and found signs of periodontal alteration years before the clinical diagnosis of periodontitis.

A recent study by Lindholm et al.^19^ evaluated the clinical and microbial profile of young patients (17 to 19 years old) with radiographic bone loss and compared them with controls without bone loss. Of 13 patients with radiographic bone loss, 12 were clinically diagnosed with periodontitis and presented higher attachment loss, probing depth, and bleeding on probing compared with the control group. After microbiological evaluation, these individuals also had higher concentrations of *A. actinomycetemcomitans*, *P. gingivalis*, and *F. alocis*, species associated with periodontitis. The fact that these findings came from patients identified by radiographic alterations increased the reliability of this diagnostic tool at this age and underscored the need for periodontal clinical evaluation when bone alterations are identified.

One of the most challenging aspects of this type of study is obtaining a high‐quality database in terms of the quantity of radiographs to permit a reliable statistical analysis and quality to guarantee the correct measurements. Using a Dental School image database, this study screened all images used for diagnosis in pediatric dentistry from almost 2500 patients and obtained 828 high‐quality bitewing radiographs. Those images represent a broad age range, from children and adolescents of both sexes treated in a public dental reference center. In addition to judicious image selection and the use of only interproximal radiographs with correct angulation, the study considered only digitally obtained radiographs, which ensures better image quality standards and reduces bias related to digitalization and alteration of the measurements. Thus, it was possible to use 2 mm as the higher limit for a normal bone level, as other similar studies that assessed bone loss in children using digital radiographs.^10^ The choice of 2 mm as a threshold for bone loss was based on most studies evaluating radiographic bone levels in children and adolescents, allowing for comparison of the results. However, some other studies have adopted CEJ‐BC > 2 mm and < 3 mm as questionable bone loss and ≥ 3 mm as definitive bone loss^12^, ^20^ to increase specificity for periodontitis cases, but at the same time, it may reduce sensitivity for detecting early bone loss.

Based on the results of the present study, the use of interproximal radiographs to evaluate the periodontal conditions in children in routine dental evaluation should be considered. This approach can be even more justifiable when children from families with a history of periodontal disease, with altered inflammatory profiles and altered local conditions are identified.^2^, ^3^, ^4^ since those aspects can potentially interfere in the susceptibility to periodontitis, and radiographs can help in disease prevention and monitoring. Additionally, periodontal probing should be routinely used jointly with radiographs as an early diagnostic tool in children since they are fundamental for clinical attachment loss diagnosis and cause no damage and minimal discomfort when adequately performed.

Despite presenting important results related to the prevalence of radiographic bone alteration in children, the lack of clinical data is an important limitation of the present study. Clinical evaluation is fundamental in correlating bone alterations with periodontal disease, and it is necessary to confirm the diagnosis of periodontitis in cases marked as positive for radiographic bone loss. However, clinical data could not be evaluated due to the nature of this study and clinical monitoring of these patients is being performed in a longitudinal study. Another critical factor is the absence of longitudinal radiographic follow‐up of the sites where bone loss was identified to confirm the presence and progression of bone loss. Therefore, it is essential that future studies clinically evaluate these children and their families and follow them up to understand the clinical and molecular factors that interfere with this process. These assessments will contribute to understand the factors that trigger periodontal losses and will also guide risk assessments and diagnosis of early periodontal losses.

## 5 CONCLUSIONS

In conclusion, this retrospective study has found a 13.28% prevalence of radiographic bone alterations in primary dentition at the patient level and 2.62% at the site level in the Brazilian population. The CEJ‐BC distance and bone loss were positively associated with local factors such as adjacent tooth exfoliation and pulp alterations; however, 41.2% of the identified bone loss sites had no association with the local risk factor evaluated in the study. Furthermore, additional clinical evaluation and longitudinal follow‐up are necessary to confirm that these alterations are associated with early signs of periodontitis.

## AUTHOR CONTRIBUTIONS

**Murilo C. Paraluppi**: Contributed to the funding acquisition; contributed to the work conception and design; contributed to data acquisition; drafted the manuscript. **Camila S. Stolf**: Contributed to data acquisition. **Bianca Mendes**: Contributed to data acquisition. **Marcio Z. Casati**: Drafted the manuscript. **Karina G. S. Ruiz**: Contributed to the work conception and design. **Enilson A. Sallum**: Contributed to data analysis. **Renato C. V. Casarin**: Contributed to the funding acquisition; contributed to the work conception and design; contributed to data analysis; drafted the manuscript. **Mabelle F. Monteiro**: Contributed to the funding acquisition; contributed to the work conception and design; contributed to data analysis; drafted the manuscript. All authors critically revised and approved the final manuscript.

## CONFLICT OF INTEREST STATEMENT

The authors declare that there are no conflicts of interest in this study.

## ETHICAL APPROVAL STATEMENT

Piracicaba Dental School ethical committee: protocol number 48784521.5.0000.5418
